# Pancolonic Dye Spray Chromoendoscopy to Detect and Resect Ill-Defined Neoplastic Lesions in Colonic Inflammatory Bowel Disease

**DOI:** 10.1093/jcag/gwac024

**Published:** 2022-08-03

**Authors:** Claudia Dziegielewski, Sarang Gupta, Jeffrey D McCurdy, Richmond Sy, Navaaz Saloojee, Sanjay K Murthy

**Affiliations:** Department of Medicine, University of Ottawa, Ottawa, Ontario, Canada; Department of Medicine, University of Toronto, Toronto, Ontario, Canada; Department of Medicine, University of Ottawa, Ottawa, Ontario, Canada; The Ottawa Hospital IBD Centre, Ottawa, Ontario, Canada; Ottawa Hospital Research Institute, Ottawa, Ontario, Canada; Department of Medicine, University of Ottawa, Ottawa, Ontario, Canada; The Ottawa Hospital IBD Centre, Ottawa, Ontario, Canada; Ottawa Hospital Research Institute, Ottawa, Ontario, Canada; Department of Medicine, University of Ottawa, Ottawa, Ontario, Canada; The Ottawa Hospital IBD Centre, Ottawa, Ontario, Canada; Department of Medicine, University of Ottawa, Ottawa, Ontario, Canada; The Ottawa Hospital IBD Centre, Ottawa, Ontario, Canada; Ottawa Hospital Research Institute, Ottawa, Ontario, Canada

**Keywords:** Colonoscopy, Chromoendoscopy, Crohn’s disease, Dye spray, Dysplasia, Inflammatory bowel disease, Neoplasia; Screening, Surveillance, Ulcerative colitis, White light endoscopy

## Abstract

**Background:**

Pancolonic dye spray chromoendoscopy (DCE) is used as an adjunct to white light endoscopy (WLE) to enhance the detection and delineation of ill-defined neoplastic (dysplastic) lesions in persons with colonic inflammatory bowel diseases (cIBD). We evaluated the utility of DCE as follow-up to high-definition WLE (HD-WLE) to “unmask” and/or facilitate endoscopic resection of neoplastic lesions.

**Methods:**

We retrospectively studied persons with cIBD who underwent DCE as follow-up to HD-WLE between 2013 and 2020. We describe neoplastic findings and management during HD-WLE and DCE exams and report outcomes from post-DCE surveillance exams.

**Results:**

Twenty-four persons were studied (mean age 56.7 ± 13.8 years, 50.0% male, 70.8% ulcerative colitis, mean disease duration 18.0 ± 11.0 years). Overall, 32 visible neoplastic lesions were unmasked during DCE, of which 24 were endoscopically resected. DCE facilitated the diagnosis of two cancers. Among 17 persons referred for evaluation of “invisible” neoplasia (detected in non-targeted biopsies) during HD-WLE, DCE identified neoplastic lesions at the same site in eight persons and a different site in four persons. Among seven persons referred for ill-defined visible neoplasia, DCE facilitated complete endoscopic resection in four individuals, whereas two individuals required colectomy for a diagnosis of cancer. Among 19 individuals with post-DCE surveillance, five developed new visible neoplastic lesions, including one high-grade neoplasia which was completely resected.

**Conclusions:**

In our cohort, DCE aided in unmasking invisible neoplasia and facilitated endoscopic resection of ill-defined neoplasia, suggesting that it is a useful surveillance tool in selected persons with cIBD. Large prospective studies are needed to validate these findings.

## BACKGROUND

Persons with colonic inflammatory bowel diseases (cIBD) have a 1.5-3-fold increased lifetime risk of developing colorectal cancer (CRC), as compared to members of the general population (1–3). As in non-IBD colons, CRCs in IBD colons develop through a series of genetic alterations that lead to progressive focal dysplastic changes in the colorectal mucosa, ultimately resulting in distinct neoplastic lesions (4). While many cancers in IBD colons develop via the adenoma-carcinoma pathway, some cancers in IBD colons develop through altered sequences of molecular events in response to chronic inflammation (4,5). This may account for some of the morphologic differences encountered in IBD-associated neoplasia, such as flat or indistinct growth patterns that blend into the surrounding mucosa, or ulcerating or stricturing growth that resembles chronic inflammatory changes (4–9). In conjunction with these observations, historical reports of high rates of synchronous and metachronous cancers in persons with neoplasia detected during WLE have led to the notion that some neoplasms may evade detection during surveillance colonoscopy until they have developed into advanced incurable cancers (10–12). Furthermore, historical studies reporting widespread genetic aberrations throughout the colitis field in IBD colons (“field cancerization”) have raised concerns about the potential for multifocal cancer development (13–16).

These observations have led to a rigorous system of colonoscopy screening and surveillance in persons with IBD, which includes application of specialized endoscopic techniques to improve neoplasia detection (6,17–19). Dye spray chromoendoscopy (DCE), in which contrast or absorptive dyes are liberally applied to the colorectal mucosa to highlight the borders, surface topography, and mucosal architecture of ill-defined neoplastic lesions (20,21), has emerged as a preferred strategy and has been recommended as a front-line modality to improve neoplasia detection in cIBD by many European and North American societies (6,19,22,23). Multiple randomized controlled trials (RCTs) have shown that DCE is superior to WLE alone for neoplasia detection in persons with cIBD (24,25). However, uptake of this technique has been limited, particularly in North America, due to issues of training, resources, and practicality (26,27). More often, DCE is reserved for a second evaluation among persons with poorly defined neoplasia identified through targeted or non-targeted sampling during WLE. However, the utility of DCE as a strategy to improve neoplasia detection and management in this setting has not been widely reported. We sought to evaluate the utility of DCE in this context in a cohort of persons with cIBD who underwent DCE as a second opinion under an endoscopist with specialized training in this technique.

## METHODS

We conducted a retrospective cohort study of consecutive adults (≥18 years old) with cIBD who underwent DCE by an endoscopist (SKM) with specialty training in this technique, over a 7-year period (September 1, 2013—December 31, 2020). We included persons who were referred as a second opinion following HD-WLE in which ill-defined neoplasia was identified, either as “invisible dysplasia” in non-targeted biopsies or as poorly defined visible irregularities in targeted biopsies. This included findings of indeterminate and definite dysplasia on biopsy. In all cases, DCE was performed within 12 months of HD-WLE, using Olympus 180 series colonoscopes (Olympus Corporation; Tokyo, Japan). DCE was performed using a standard approach of aggressive cleansing of the colonic mucosa during endoscope insertion and application of 0.04% methylene blue in sterile water to the entire colonic mucosal surface during withdrawal of the colonoscope using the Erbe waterjet system (ERBE Elektromedizin, GmbH; Tubingen, Germany) (20,28). Suspicious mucosal irregularities highlighted by white light or dye were either resected or biopsied. Non-targeted biopsies were routinely taken from areas of poor visibility (due to inflammation, scarring, or post-inflammatory polyps).

We collected demographic and disease-specific characteristics for all individuals. Baseline characteristics included age, sex, IBD type (Crohn’s disease [CD] vs. ulcerative colitis [UC]), IBD duration, IBD phenotype, maximum colitis severity (historical), history of colorectal surgery, personal history of dysplasia, history of primary sclerosing cholangitis (PSC) and family history of CRC. We classified disease phenotype using the Montreal classification (29) and colitis severity using the Mayo endoscopic subscore (30).

We collected information on the number, location and type (visible or invisible) of neoplastic lesions during the initial HD-WLE exam and follow-up DCE exam for each person. Neoplastic lesions were reported as adenomas, serrated lesions (with or without dysplasia) or non-specific dysplasia (usually when identified by non-targeted biopsies). Dysplasia grade was reported as indeterminate, low-grade (default for adenomas), high-grade or CRC, and confirmed by at least two gastrointestinal pathologists. We reported the highest grade of neoplasia for each procedure. We counted each invisible neoplasia detected by a non-targeted biopsy as a unique focus and each unique area of visible neoplasia (irrespective of the number of targeted biopsies) as a unique focus. We documented whether endoscopic resection was attempted and whether resection was considered complete for each visible neoplastic lesion. We observed individuals until December 31, 2020, for the development of colorectal neoplasia during subsequent surveillance colonoscopies.

We report neoplastic findings during the two examinations (HD-WLE and DCE) and descriptive summary statistics, with percentages, medians (with interquartile range) or means (with standard deviation), as appropriate. Study approval was granted by The Ottawa Health Sciences Network Research Ethics Board.

## RESULTS

### Person Characteristics

A total of 24 DCE exams in 24 persons met study criteria. Person characteristics are reported in Supplementary Table 1. There were 12 males (50.0%) and 17 persons (70.8%) with UC. The mean age was 39.6 ± 14.3 years at the time of IBD diagnosis and 56.7 ± 13.8 years at the time of DCE. The mean disease duration was 18.0 ± 11.0 years, and 17 persons (70.8%) had at least moderate maximal disease severity. There were 10 persons (41.7%) with a prior history of dysplasia, 2 (8.33%) with partial colectomy, 1 (4.17%) with PSC, and 3 (12.5%) with CRC in a first-degree relative.

### Neoplastic Detection During HD-WLE and DCE Exams

Procedural findings during HD-WLE and DCE exams are listed in Supplementary Table 2. The mean time between HD-WLE and DCE was 7.50 ± 6.14 months. The indication for DCE referral in 17 persons (70.8%) was invisible neoplasia identified through non-targeted biopsies, and in 7 persons (29.2%) was poorly defined visible neoplasia to survey the rest of the colon and assist in dysplasia delineation and management. During the index HD-WLE exams, 12 visible neoplastic lesions were identified in 10 persons (two of which were not resected) and 26 invisible neoplastic foci were identified through non-targeted biopsies in 17 persons.

In total, 34 visible neoplastic lesions were identified in 17 persons using DCE, including 32 unique lesions that were not identified during the preceding HD-WLE exams. These lesions were found in the ascending colon (11), transverse colon (11), descending colon (1), sigmoid colon (5) and rectum (6). The histologic findings included 17 tubular adenomas, 2 tubulovillous adenomas, 8 sessile serrated lesions, 5 unspecified dysplasia and 2 carcinomas (identified as pre-cancerous dysplasia on WLE). Overall, DCE helped visualize an additional 1.33 neoplastic lesions per person and delineated 2 ill-defined lesions that were misdiagnosed as pre-cancerous dysplasia during prior HD-WLE, permitting diagnosis of CRC in both lesions.

Among the 17 persons who had invisible neoplasia identified during HD-WLE, visible neoplasia was identified at the same anatomic location in eight persons (47.1%) and at a different anatomic location in four persons (23.5%) using DCE. Discrete neoplasia was not unmasked in five persons with prior invisible neoplasia (29.4%).

Among the seven persons referred for further management of ill-defined visible neoplasia identified during HD-WLE, nine neoplastic lesions were visualized in five persons using DCE. The other two persons had no visible dysplasia identified on DCE.

Additionally, of non-targeted biopsies that were obtained during DCE exams from areas of significant scarring or inflammation, two invisible neoplastic foci were identified in two individuals.

### Neoplasia Management During DCE

Endoscopic resection was successfully carried out for 24 of 34 (70.6%) visible neoplastic lesions identified during DCE.

Among the 17 persons referred for DCE for invisible neoplasia, complete resection of identified visible lesions was carried out in 12 persons (70.5%). Among the other five individuals, three had biopsy confirmation of dysplasia identified at the previous site but did not have identifiable lesions to resect, while two others had challenging surveillance due to pseudopolyposis and scarring and therefore discrete dysplastic lesions were difficult to resect.

Among seven persons referred for ill-defined visible dysplasia, resection was completed during DCE exams in four persons (57.1%). Of those with incomplete dysplasia resection, two underwent colectomy for an ultimate diagnosis of CRC, and one had submucosal fibrosis which necessitated referral to an advanced therapeutic endoscopist for attempted resection.

### Follow-Up After DCE

A total of 49 follow-up colonoscopies were performed in 19 individuals in our cohort following DCE (median follow-up time 20.5 [13.2–25.8] months; mean total observation time 581 ± 356 months). Within this group, 11 persons (57.9%) did not develop new neoplasia, 5 individuals (26.3%) developed new neoplasia and 3 (15.8%) individuals had persistent neoplasia identified on prior DCE.

For the five individuals who developed new neoplasia (time to first neoplasia 30.8 ± 11.6 months), there were 11 new visible neoplastic lesions and 40 invisible neoplastic foci (in non-targeted biopsies). Endoscopic resection was complete for 10/11 (90.9%) visible neoplastic lesions; in 1 individual, they required serial colonoscopy to complete resection of a polypoid cluster with low-grade neoplasia. Of these five individuals, three developed low-grade neoplasia at the same site of prior neoplasia, one developed low-grade neoplasia at a different site, and one developed visible high-grade neoplasia at a different site (completely resected endoscopically). Importantly, the individual who was found to have high-grade neoplasia had complete endoscopic resection of this lesion and was only found to have indeterminate neoplasia on the final follow-up exam.

For the three individuals with persistent neoplasia previously identified on colonoscopy, there were five visible lesions and 27 invisible lesions. Two of these persons had persistent invisible neoplasia in the context of challenging surveillance due to pseudopolyposis and scarring, and one had visible neoplasia with complex lesions characteristics; the latter ultimately underwent attempted lesion resection under an advanced therapeutic endoscopist. No CRC was identified in any of the individuals who underwent follow-up colonoscopy.

## DISCUSSION

In this retrospective study of 24 individuals with cIBD referred to an endoscopist with expertise in DCE to further evaluate invisible neoplasia or ill-defined visible neoplasia detected during HD-WLE, an additional 32 previously unidentified visible neoplastic lesions were unmasked during DCE, for an average of 1.33 additional visible neoplastic lesions per person. Among individuals referred for evaluation of invisible neoplasia identified through non-targeted biopsies, more than 70% had visible lesions unmasked during DCE, although lesions were not detected in more than 50% of persons from the initial site of invisible neoplasia. Importantly, no individuals with invisible neoplasia who did not have lesions unmasked by DCE developed advanced neoplasia during subsequent surveillance, suggesting that these individuals had minute foci of low-grade neoplasia that were clinically irrelevant. DCE further unmasked and permitted complete endoscopic resection of visible neoplastic lesions in more than half of persons referred for ill-defined visible abnormalities and facilitated a diagnosis of carcinoma in two other individuals in this group who were misdiagnosed as having pre-cancerous dysplasia. In both of the latter cases, DCE permitted much better appreciation of the full breadth of these lesions, which improved lesion sampling and led to a more accurate diagnosis. In persons with evaluable follow-up colonoscopy data, one-quarter developed new visible neoplastic lesions, almost all of which were completely resected endoscopically. Overall, DCE unmasked and/or facilitated endoscopic resection of visible lesions in most of the individuals in this cohort, and no individuals who continued in endoscopic surveillance developed high-risk lesions that were not manageable endoscopically.

To the best of our knowledge, this is the first study to showcase the utility of DCE in this setting. While further studies are required to support our findings, the observations lend credibility to the utility of DCE as a follow-up to HD-WLE in persons with ill-defined neoplasia. Our findings build on previous reports of higher neoplasia detection rates with DCE as compared to WLE (4–6,16). However, with waning benefits of DCE for neoplasia detection in the era of HD-WLE (24) and newer generation “click of a button” narrow band imaging (31,32), the value of DCE as a front line surveillance modality is questionable (6,33,34). Nonetheless, our findings support a role for DCE as a secondary surveillance modality in individuals with complex or ill-defined dysplasia. In line with this, updated guidance from the American Gastroenterological Association states that “… a finding of invisible dysplasia should prompt repeat examination by an experienced endoscopist using high-definition dye spray chromoendoscopy…” and “…for visible lesions that can be resected or if histologic dysplasia is not confirmed on a high-quality dye spray chromoendoscopy examination, continued endoscopic surveillance at frequent intervals is appropriate…” (6). Notably, this expert review also recommends that “…a finding of unresectable visible dysplasia or of invisible multifocal or high-grade dysplasia on histology during DCE should prompt colectomy.”

The frequent finding of visible neoplasia during DCE at sites of prior invisible neoplasia during HD-WLE supports the notion that many such lesions are not truly invisible, but may simply go undetected without highly sensitive imaging modalities (6). Additionally, the ability of DCE to facilitate complete resection of visible lesions in more than half of individuals referred for poorly-defined lesions during WLE supports a role for dye spray to highlight lesion borders and surface topography. Furthermore, the absence of visible neoplasia during DCE in more than a quarter of individuals referred for a finding of invisible neoplasia, with no high-risk neoplasia developing in these individuals during long-term follow-up, is reassuring and supports a role for continued surveillance with DCE over colectomy in some persons with invisible dysplasia. Some of these individuals may have had microscopic foci of neoplasia that were removed in their entirety with biopsy forceps or that simply did not evolve to more significant lesions with time. Others may have had neoplastic-type mucosal architectural changes (but not true neoplasia) due to the inflammatory milieu (6). Additionally, there may be uncertainty of dysplasia diagnosis due to pathologist inter-observer variability, as well as poor endoscopic-histologic correlation, which may lead to an overcall of invisible dysplasia not otherwise detectable by DCE (35,36). However, as several individuals in our cohort developed new neoplastic lesions during follow-up exams, a “normal” or reassuring DCE exam does not obviate the need for continued close surveillance with DCE with non-targeted biopsies taken around sites of prior invisible and visible neoplasia, as supported by recent guidelines (6).

There are several limitations to this study. Firstly, this is a retrospective single-centre, single investigator, study in a small cohort of persons, limiting the precision of our findings as well as generalizability to the broader IBD population. Thus, our findings require validation in larger cohorts of individuals with cIBD from other centers. Secondly, indications for referral and biopsy patterns during HD-WLE were not protocolized, which may have affected neoplasia rates during HD-WLE and DCE exams. In addition, there was a time difference between initial neoplasia diagnosis and DCE, which may have contributed to initially invisible lesions becoming visible due to lesion growth, although the timeframe is quite short. Also, we did not have a control group of persons who underwent repeat HD-WLE to confirm that it was indeed dye spray that led to improved neoplasia detection and management and not simply a repeat high-quality colonoscopy by another endoscopist. Finally, we would require longer-term follow-up data to determine neoplasia and cancer rates many years following DCE in our cohort, which would provide further insight into the clinical utility of DCE in persons with cIBD.

## CONCLUSIONS

In our retrospective cohort, we observed DCE to be a highly useful tool to unmask and treat ill-defined neoplasia identified during HD-WLE in persons with cIBD. We recommend close surveillance with DCE by a trained endoscopist in this high-risk setting while reserving colectomy for persons with very high-risk findings (i.e., unresectable visible neoplasia or invisible multifocal, high-grade or recurrent neoplasia despite DCE). Further prospective studies in larger cohorts of individuals with long-term follow-up are required to validate our findings.

## Supplementary Material

gwac024_suppl_Supplementary_Tables
