# Supplementary material for: Pancolonic Dye Spray Chromoendoscopy to Detect and Resect Ill-Defined Neoplastic Lesions in Colonic Inflammatory Bowel Disease
Source: J Can Assoc Gastroenterol. 2022 Aug 3;6(1):37–41. doi: 10.1093/jcag/gwac024 (PMC9915055; doi:10.1093/jcag/gwac024)
Supplement: gwac024_suppl_Supplementary_Tables [file gwac024_suppl_supplementary_tables.docx]

**Supplementary File**

**Pancolonic Dye Spray Chromoendoscopy to Detect and Resect Ill-Defined Neoplastic Lesions in Colonic Inflammatory Bowel Disease**

Claudia Dziegielewski^1^ M.D., Sarang Gupta^2^ M.D., Jeffrey D. McCurdy^1,3,4^ M.D., FRCPC, Ph.D., Richmond Sy^1,3,4^ M.D., FRCPC, Navaaz Saloojee^1,4^ M.D., FRCPC, Sanjay K. Murthy^1,3,4^ M.D., FRCPC, M.Sc.

^1^Department of Medicine, University of Ottawa, Ottawa, ON, Canada;

^2^Department of Medicine, University of Toronto, Toronto, ON, Canada;

^3^Ottawa Hospital Research Institute, University of Ottawa, Ottawa, ON, Canada

^4^The Ottawa Hospital IBD Centre, Ottawa, ON, Canada

Corresponding author: Sanjay K. Murthy, Department of Medicine, University of Ottawa, 501 Smyth Road, Ottawa, ON, Canada, K1H 8L6. Ph: 613-737-8899; Email: [smurthy@toh.ca](mailto:smurthy@toh.ca)

**Key Words:** Inflammatory bowel disease, ulcerative colitis, Crohn’s disease, screening, surveillance, colonoscopy, chromoendoscopy, dye spray, white light endoscopy, dysplasia, neoplasia

**Table 1:** Baseline characteristics in persons with colonic inflammatory bowel diseases who underwent HD-WLE followed by DCE.

| **Baseline Characteristics** | | | | | | | | | | | |
| --- | --- | --- | --- | --- | --- | --- | --- | --- | --- | --- | --- |
| **ID** | **Sex** | **Age** | **IBD Type** | **Duration of disease** | **Montreal classification** | **Maximum disease severity** | **History of colon resection** | **History of neoplasia** | **History of PSC** | **Family history of CRC** |  |
| **1** | M | 64 | CD | 9 | A3L2B1 | Moderate | No | No | No | No |  |
| **2** | M | 51 | CD | 15 | A3L2B1 | Moderate | Yes | No | No | No |  |
| **3** | F | 65 | UC | 35 | E3 | Severe | No | Yes | No | Yes |  |
| **4** | F | 61 | UC | 18 | E2 | Severe | No | No | No | No |  |
| **5** | F | 43 | UC | 2 | E3 | Moderate | No | Yes | Yes | No |  |
| **6** | F | 71 | UC | 30 | E3 | Mild | No | No | No | No |  |
| **7** | F | 73 | CD | 45 | A2L3B2 | Moderate | No | No | No | No |  |
| **8** | M | 40 | CD | 23 | A2L2B1p | Moderate | No | No | No | No |  |
| **9** | M | 45 | UC | 22 | E3 | Moderate | Yes | No | No | No |  |
| **10** | F | 63 | UC | 31 | E2 | Mild | No | Yes | No | No |  |
| **11** | M | 35 | UC | 13 | E3 | Mild | No | No | No | No |  |
| **12** | F | 35 | CD | 16 | A2L2B1 | Moderate | No | No | No | Yes |  |
| **13** | M | 65 | CD | 10 | A3L2B1p | Severe | No | Yes | No | No |  |
| **14** | M | 75 | CD | 16 | A3L3B1 | Mild | No | No | No | No |  |
| **15** | M | 38 | UC | 18 | E2 | Moderate | No | Yes | No | No |  |
| **16** | M | 61 | UC | 7 | E3 | Mild | No | Yes | No | No |  |
| **17** | M | 64 | UC | 12 | E3 | Mild | No | No | No | Yes |  |
| **18** | M | 64 | UC | 15 | E3 | Moderate | No | No | No | No |  |
| **19** | F | 78 | UC | 7 | E3 | Moderate | No | Yes | No | No |  |
| **20** | M | 74 | UC | 37 | E3 | Moderate | No | No | No | Yes |  |
| **21** | F | 44 | UC | 11 | E2 | Moderate | No | Yes | No | No |  |
| **22** | F | 53 | UC | 3 | E2 | Moderate | No | Yes | No | No |  |
| **23** | F | 39 | UC | 13 | E2 | Moderate | No | Yes | No | No |  |
| **24** | F | 60 | UC | 24 | E3 | Mild | No | No | No | No |  |

**Notes**: Duration of disease is time from IBD diagnosis to time at DCE exam. History of neoplasia is neoplasia on colonoscopies prior to the latest HD-WLE colonoscopy preceding DCE. For patients with a family history of CRC, ID #3 had a brother and sister diagnosed at age 50, ID #12 had a father diagnosed at age 39, ID #17 had a mother diagnosed at age 80, and ID #20 had a brother diagnosed at age 50. M=male, F=female, CD=Crohn’s disease, UC=ulcerative colitis, CRC=colorectal cancer.

**Table 2**: Procedural findings in persons with colonic inflammatory bowel diseases who underwent HD-WLE followed by DCE.

|  | **HD-WLE** | | | | | | | **DCE** | | | | | |
| --- | --- | --- | --- | --- | --- | --- | --- | --- | --- | --- | --- | --- | --- |
| **ID** | **Bowel preparation** | **Mucosal inflammation** | **# Visible neoplastic lesions** | **Type of neoplastic lesions** | **# Invisible lesions** | **Highest grade of neoplasia** | **Reason for DCE referral** | **Bowel preparation** | **Mucosal inflammation** | **# Visible neoplastic lesions** | **Type of neoplastic lesions** | **Highest grade of neoplasia** | **DCE findings** |
| **1** | Good | None | 1 | Pedunculated (Ip) | 1 | LGN | Invisible neoplasia | Excellent | None | 2 | Pedunculated (Ip) x2 | LGN | New visible neoplasia in site of invisible dysplasia; incomplete resection |
| **2** | Good | Moderate (left colon) | 1 | Pedunculated (Ip) | 1 | LGN | Invisible neoplasia | Excellent | Moderate (rectum, left colon) | 3 | Pedunculated (Ip) x2, flat elevated (IIa) x1 | LGN | New visible neoplasia in site of invisible dysplasia; complete resection |
| **3** | Excellent | Mild (right colon) | 2 | Flat elevated (IIa) x2 | 0 | LGN | Visible neoplasia / assist resection | Excellent | None | 1 | Flat elevated (IIa) | LGN | New visible neoplasia in site of prior visible dysplasia; complete resection |
| **4** | Excellent | None | 1 | Flat elevated (IIa) | 0 | LGN | Visible neoplasia / assist resection | Excellent | Mild (rectum) | 0 | N/A | N/A | No neoplasia identified |
| **5** | Good | Mild (patchy) | 2 | Pedunculated (Ip) x2 | 0 | LGN | Visible neoplasia / assist resection | Good | Mild (right colon) | 0 | N/A | N/A | No neoplasia identified |
| **6** | Good | None | 1 | Pedunculated (Ip) | 2 | LGN | Invisible neoplasia | Excellent | None | 2 | Sessile (Is) x2 | LGN | New visible neoplasia in site of prior invisible dysplasia; biopsied but no attempt to resect |
| **7** | Excellent | Mild (TI) | 0 | N/A | 1 | LGN | Invisible neoplasia | Excellent | None | 1 | Pedunculated (Ip) | LGN | New visible neoplasia in other site, not at site of invisible dysplasia; complete resection |
| **8** | Good | Mild (right colon) | 0 | N/A | 0 | None | Visible neoplasia / assist resection | Excellent | None | 1 | Pedunculated (Ip) | LGN | Partial resection of visible lesion |
| **9** | Good | None | 1 | Flat elevated and depressed (IIa+c) | 0 | LGN | Visible neoplasia / assist resection | Excellent | None | 1 | Flat elevated and depressed (IIa+c) | CRC | Could not resect lesion |
| **10** | Excellent | None | 0 | N/A | 1 | LGN | Invisible neoplasia | Excellent | Mild (left colon) | 1 | Sessile (Is) | LGN | New visible neoplasia in other site, not at site of invisible dysplasia; complete resection |
| **11** | Excellent | Mild (pancolitis) | 1 | Flat elevated (IIa) | 0 | LGN | Visible neoplasia / assist resection | Excellent | None | 1 | Flat elevated (IIa) | LGN | New visible neoplasia in other site, not at site of prior visible dysplasia; complete resection |
| **12** | Excellent | Mild (patchy) | 0 | N/A | 4 | LGN | Invisible neoplasia | Excellent | Mild (patchy) | 1 | Flat (IIb) | Indet. | New visible neoplasia in site of prior invisible dysplasia; biopsied nodular area, no attempt to resect |
| **13** | Good | None | 0 | N/A | 3 | LGN | Invisible neoplasia | Fair | None | 3 | Pedunculated (Ip) x2, sessile (Is) x1 | LGN | New visible neoplasia in site of invisible dysplasia; complete resection |
| **14** | Good | None | 0 | N/A | 1 | LGN | Invisible neoplasia | Excellent | None | 0 | N/A | N/A | No neoplasia identified |
| **15** | Excellent | Mild (rectum) | 0 | N/A | 3 | LGN | Invisible neoplasia | Fair | Moderate (rectum) | 5 | Flat elevated (IIa) x3, flat elevated and depressed (IIa+c) x2 | LGN | New visible neoplasia in other site, not at site of prior invisible dysplasia; complete resection |
| **16** | Poor | Mild (left colon) | 0 | N/A | 1 | LGN | Invisible neoplasia | Excellent | Mild (patchy) | 1 | Flat elevated (IIa) | LGN | New visible neoplasia in other site, not at site of prior invisible dysplasia; complete resection |
| **17** | Good | Mild (rectum) | 0 | N/A | 1 | LGN | Invisible neoplasia | Excellent | Mild (rectum) | 0 | N/A | N/A | No neoplasia identified |
| **18** | Excellent | None | 1 | Pedunculated (Ip) | 1 | LGN | Invisible neoplasia | Excellent | None | 0 | N/A | N/A | No neoplasia identified |
| **19** | Good | None | 0 | N/A | 1 | LGN | Invisible neoplasia | Excellent | None | 0 | N/A | N/A | No neoplasia identified |
| **20** | Good | None | 0 | N/A | 1 | LGN | Invisible neoplasia | Good | None | 3 | Pedunculated (Ip) x3 | LGN | New visible neoplasia in site of invisible dysplasia; complete resection |
| **21** | Good | Moderate (pancolitis) | 0 | N/A | 2 | LGN | Invisible neoplasia | Excellent | Moderate (left colon) | 1 | Flat elevated (IIa) | LGN | New visible neoplasia in site of invisible dysplasia; partial resection |
| **22** | Good | Mild (left colon) | 0 | N/A | 1 | LGN | Invisible neoplasia | Excellent | Mild (left colon) | 2 | Pedunculated (Ip) x1, flat (IIb) x1 | LGN | New visible neoplasia in site of invisible dysplasia; complete resection |
| **23** | Excellent | Moderate (left colon) | 1 | Pedunculated (Ip) | 0 | HGN | Visible neoplasia / assist resection | Excellent | None | 5 | Flat elevated (IIa) x3, pedunculated (Ip) x2, | CRC | New visible neoplasia in site of prior visible dysplasia; complete resection for polyps not for CRC |
| **24** | Excellent | Mild (rectum) | 0 | N/A | 1 | LGN | Invisible neoplasia | Excellent | Mild (rectum) | 0 | N/A | N/A | No neoplasia identified |

Notes: Mucosal inflammation is described at the time of endoscopy, and the location in which it was present. Visible neoplastic lesions are classified according to the Paris classification^1^. Highest grade of neoplasia is the maximum grade of neoplasia for either visible or invisible dysplastic lesions. LGN=low grade neoplasia, Indet.=indeterminate, HGN=high grade neoplasia, CRC=colorectal cancer, N/A=not applicable.

^1^ Murthy SK, Feuerstein JD, Nguyen GC et al. AGA clinical practice update on endoscopic surveillance and management of colorectal dysplasia in inflammatory bowel diseases: Expert review. Gastroenterology 2021;161(3):1043-51.e4.
